# Artificial Cardiac Muscle with or without the Use of Scaffolds

**DOI:** 10.1155/2017/8473465

**Published:** 2017-08-10

**Authors:** Yifei Li, Donghui Zhang

**Affiliations:** ^1^Department of Pediatrics, West China Second University Hospital, Sichuan University, Chengdu, China; ^2^Key Laboratory of Ministry of Education for Women and Children's Diseases and Birth Defects, West China Second University Hospital, Sichuan University, Chengdu, China; ^3^Department of Cardiology, Boston Children's Hospital, Boston, MA, USA; ^4^Hubei Collaborative Innovation Center for Green Transformation of Bio-Resources, College of Life Sciences, Hubei University, Wuhan, China

## Abstract

During the past several decades, major advances and improvements now promote better treatment options for cardiovascular diseases. However, these diseases still remain the single leading cause of death worldwide. The rapid development of cardiac tissue engineering has provided the opportunity to potentially restore the contractile function and retain the pumping feature of injured hearts. This conception of cardiac tissue engineering can enable researchers to produce autologous and functional biomaterials which represents a promising technique to benefit patients with cardiovascular diseases. Such an approach will ultimately reshape existing heart transplantation protocols. Notable efforts are accelerating the development of cardiac tissue engineering, particularly to create larger tissue with enhanced functionality. Decellularized scaffolds, polymer synthetics fibrous matrix, and natural materials are used to build robust cardiac tissue scaffolds to imitate the morphological and physiological patterns of natural tissue. This ultimately helps cells to implant properly to obtain endogenous biological capacity. However, newer designs such as the hydrogel scaffold-free matrix can increase the applicability of artificial tissue to engineering strategies. In this review, we summarize all the methods to produce artificial cardiac tissue using scaffold and scaffold-free technology, their advantages and disadvantages, and their relevance to clinical practice.

## 1. Introduction

Cardiovascular disease is one of the most severe health problems in the world, and it leads to the highest number of mortalities every year. Moreover, rapid changes in social and living patterns over the past several decades have made cardiovascular disease the most important challenge worldwide. Typically, heart failure (HF) is the final common stage of most types of cardiovascular disease, preceded by myocardial infarction (MI), hypertension, arrhythmia, and a variety of cardiomyopathies [1]. According to several clinical observations, the 5-year survival rate of late stage HF is similar to that of some types of cancer [1], which is approximately 45–60% [2]. Pathological cardiac remodeling occurs before HF during the pathophysiological process. Such remodeling changes the contractile proteins from adult to fetal isoforms, and the conversion of fatty acid oxidation (FAO) to glycolytic metabolism was identified along with HF, regardless of underlying etiologies. Through a great deal of effort made since the 1960s, treatments of *β*-blockers, angiotensin converting enzyme inhibitors (ACEI), and angiotensin receptor blocker (ARB) are taken as additive therapeutic methods as cardiotonic and diuretic medication strategies for long-term HF treatment [3]. However, due to the very limited endogenous capacities resident cardiomyocytes proliferation and regeneration in adult heart tissue, current strategies to improve and prolong the patient's life can only help alleviate the symptoms and slow the pathological process of HF. To date, whole heart transplantation is the most efficient therapy for HF, but it is usually trapped and challenged by ethical issues, donor availability, long-term immunosuppressant treatment, and complex surgical processes for waiting recipients.

Despite this, cardiac tissue engineering offers the possibility of restoring the contractile function and retain the pumping features of the human heart. The aim of tissue engineering is to develop autologous and functional biomaterials that can be implanted into the injured tissues. Based on this concept, cardiac tissue engineering has been introduced as a promising technique to benefit patients with cardiovascular disease. Moreover, several notable efforts have been made to move cardiac regeneration forward [4]. Artificial cardiac tissue engineering has evolved, and the ambition of this technique is to develop the entire organ from one cell, rather than only creating a small part of tissue. Successfully engineered tissue always follows a well-established design paradigm, which includes three primary components [4–6]: (1) a scaffold or an environment that provides a three-dimensional structure for tissue growth; (2) cells seeded or cultured on the scaffold or environment; (3) humoral and mechanical signaling that directs de novo tissue formation and host remodeling. The production protocols to create autologous functional tissues are as diverse as the potential applications of tissue engineered implants and are therefore difficult to encompass in a single discussion. However, the scaffold is one of the most important components involved in the tissue formation, so the optimal applications of scaffolds can be a key point of a successful strategy. At the same time, a scaffold-free protocol has been developed that is much closer to clinical practice, follows different treatment objectives, and is challenging the concept of the scaffold. According to recent research results, scaffold and scaffold-free protocols display unique advantages and disadvantages, making it difficult to identify which is the superior technique. This review focuses on the specific priorities of scaffold and scaffold-free applications in cardiovascular tissue engineering with respect to the myocardium and the whole heart.

## 2. Scaffold: Pacing to Functional Artificial Tissue

At the very beginning of cardiac tissue engineering, the idea of the injection of multipotent cells into the infarcted myocardium, termed cellular cardiomyoplasty, was developed as an alternative therapeutic strategy for end stage HF or cardiomyopathy [7–9]. A series of studies reported such techniques could markedly improve specific heart functions with regenerated pieces of tissue following cell implantation from mesenchymal stem cells (MSCs) [10, 11], embryonic stem cells (ESCs) [12, 13], and induced pluripotent stem cells (iPSCs) [14, 15]. However, problems with this strategy arose from the unexpectedly low cellular retention rate, likely due to poor nutritional supplementation and the lack of a proper matrix.

A pioneering study proved that an optimized microscale architecture culture system with patterns of cells in small cell clusters could improve survival and maintenance of function, assessed by gene expression profiles and physiological metabolism [20]. Subsequently, a different matrix providing various modes of elasticity, was shown to induce MSCs differentiation into separated shapes [21–25]. In line with these findings, a model to comprehensively investigate the effect of matrix elasticity on myocyte contractility has been developed. Such results suggested that the matrix elasticity dominates the intracellular elasticity and that the function of the myocytes was related to its aspect ratios and the elasticity of the extracellular matrix [26]. It was indicative that artificially patterning stem cells by mimicking the appropriate elasticity of the matrix or natural cell shape could facilitate functional maturation. To date, the scaffold is widely accepted to play an important role because it not only supports the survival of transferred cells, but also shapes their function, a significant milestone in tissue engineering.

**Figure 1:**
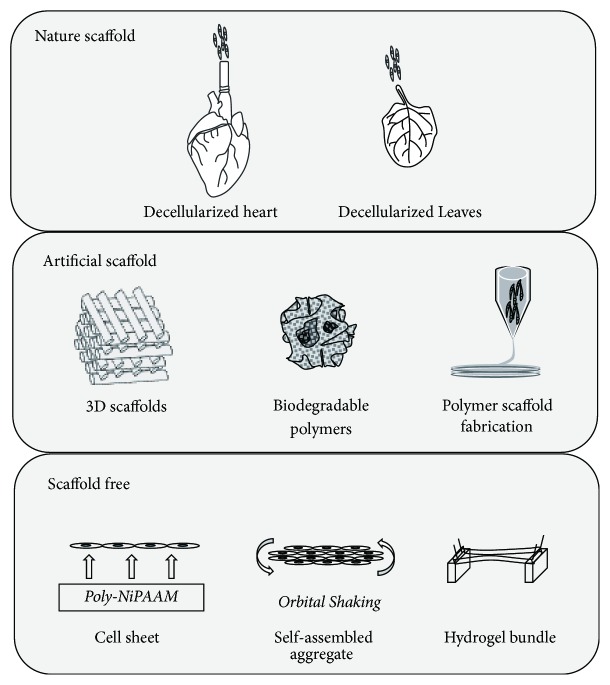
The strategies to build artificial cardiac muscle.

One strategy has been developed to make 3-dimensional (3D) patches of implantable biomaterials. Commonly, the methods for building 3D patches are the “scaffold” and “scaffold-free” approaches. The scaffold-based approach uses the original scaffold from an in vivo matrix to produce an artificial scaffold (Figure 1). Under such circumstances, bioengineered scaffolds, or “cardiac patches,” provide physical support for the myocardium, while maintaining transplanted cells survival and physiological properties via mechanical stress. In this regard, the most straightforward strategy is to use decellularized matrix from primary tissue, where removal of all live cells leaves only the underlying structure. Over the past decade, a variety of decellularized protocols, including tissue- or organ-specific, physical, chemical, and enzymatic methods, have been successfully developed [27]. Moreover, the rapid development of biomaterials has increased the amount of sources for building scaffolds. Natural biomaterials such as collagen, fibrin, or alginate are the most basic and popular source materials for synthetic scaffolds with acceptable biocompatibilities. Common artificial polymer synthetics that include poly(*ε*-caprolactone) (PCL), polylactic acid (PLA), or polyethylene glycol (PEG) were also used to build biological scaffolds with the quick development of industrial polymer-producing technologies [28, 29]. Compared to natural materials, synthetic polymers are easier to process but are usually less biocompatible [30]. However, the advantage of microfabrication techniques allows the formation of unique structures from synthetic polymers, which could be more suitable for the repair of a specific injured area. Additionally, such methods could recapitulate the morphological and physiological patterns of natural tissues, thereby enabling implantation with enhanced biological function performance [31]. An important proof-of-concept study based on porcine models of MI demonstrated improved myocardial neovascularization and improved LV contractility after implantation of fibrin patches seeded with ESCs derived from smooth muscle and endothelial cells [32]. Some specific scaffolds were quite close to clinical practice as several clinical trials were launched, including the MAGNUM trial with collagen patches [17] and the PRESERVATION I trial using bioabsorbable sodium alginate and calcium gluconate scaffolds [18]. All of the approaches to achieve the 3D matrix for cardiac tissue repair are summarized in Figure 1.

Because scaffolds are considered a key element in making artificial organs more functional, manufacturing applicable matrix and scaffolds efficiently remains one of the most important factors in cardiac tissue engineering. With the rapid development of nanotechnology and biomaterials, several novel methods have been implemented to generate cardiac scaffolds, including bioprinting, electrospinning, lithography, dielectrophoresis, and microfluidics. Bioprinting is a type of creative manufacturing for building two-dimensional (2D) or 3D structures based on biological objects using the appropriate resolution, mechanical properties, and various types of materials. With the help of computer-assisted manufacturing, the bioprinting technique can build scaffolds from natural materials and synthetic polymers with precise size and structure, which is required for patient transplants and modeling heart patterns. Bioprinting is useful for developing a macrostructure of cardiac scaffolds but fails when building a very tiny structure. The technique should be handled by experts, with wide range control of the scaffold design, including the pore size, biodegradability, and biocompatibility. Another technique is electrospinning, which only uses artificial polymers, such as PLA, PCL, and PEG. This type of technique is useful to make a microstructure with high resolution, in direct contrast to bioprinting. However, artificial materials provide only high stiffness, which might not fit all types of tissue engineering and might be limited to several types of cells requiring more dynamic matrix properties. Additionally, such scaffolds cannot provide unlimited extension and growth space necessary for cell seeding. Alternative methods include lithography and dielectrophoresis, two easy to handle techniques used to create micropatterns at low costs. Both can help cardiomyocytes pattern in singular or multiple layer(s). Although it provides a 3D multicellular culture environment system, it only results in path-like tissue, which is not useful for larger tissue repairs. Finally, the microfluidic platform is a method which provides a 3D multicellular culture system within a dynamic environment, allowing researchers to produce complex microdevices. Although easy to operate, different microfluidic platforms are required to make different scaffolds, and the surface chemistry of the substrate is a major design consideration prior to building the scaffold. As demonstrated here, there are several advanced methods for creating scaffolds, each of which has its own advantages and disadvantages for cardiac tissue engineering. Thus, choosing the best approach to obtain the desired scaffold still plays an important role in preparing functional cardiac tissue.

## 3. Decellularized Scaffolds

Decellularized scaffolds are derived from underlying structures from cadaveric or animal sources via cell removal. This is followed by reseeding or implantation alone to rebuild tissues and organs. In theory, the decellularized scaffolds of allogeneic or xenogeneic tissues will retain natural fibrous aspects with three-dimensional structure, which provides a framework for a specific organ [33]. Preserved architecture and mechanical properties are essential for more direct applications of the reconstituted tissue. The physiological cellular support platform provides seeded cells a suitable microenvironment to direct their proliferation, adhesion, migration, differentiation, and survival. Thus, such physiological structures provide the best biocompatibility among existing scaffolds. Yet, the decellularization technique is only successful if the transplanted architecture survives. This is complicated by the risk of antigenicity and pathogen transmission posed by decellularized tissue.

To date, a series of tissues and organs, including myocardium, pericardium, heart valves, lungs, liver, bone, and vasculature, were used for this type of scaffold preparation [34–37]. Physical, chemical, and enzymatic methods are administered to decellularize tissues [38, 39], and extra effort is placed on removing the maximal amount of native cells from the scaffolds. This intends to avoid the risk of rejection, both for the host recipient and for the reseeded cells to proliferate and populate available scaffold space (Table 1) [40].

Decellularized tissues were first manufactured in small pieces for minimal transplantation. In the case of the heart, tissues were directly inserted into the infarcted myocardium to preserve heart function. Additionally, the myocardium extracellular matrix (ECM) offered physiological properties similar to the microenvironment to the natural myocardium making it the most common choice for cardiac tissue engineering. Myocardium ECM scaffolds were applied in several studies in vivo to test whether they could improve the function of injured hearts. Dai et al. conducted a study to locally implant decellularized myocardial ECM scaffold into infarcted cardiac regions in a rat model [41]. An enhanced contractile function with 8% elevation in the left ventricular ejection faction (LVEF) and a 1.2-fold increase in thickness were observed, along with a reduction of the injured zone. Similar results were identified in other studies. Following injection of decellularized myocardial ECM scaffold into damaged cardiac tissue, studies in a rat model reported preserved ejection fraction, increased viable myocardium islands inside the infarct zone, and elevated proliferative cell density [42], findings corroborated in porcine models [43]. Moreover, these promising results encouraged further studies researching the combination of decellularized ECM scaffolds and fibrin with implanted mesenchymal progenitor cells, ultimately demonstrating results leading to clinical transition [44]. Further, multiple sources reported forming complex scaffolds rooted on decellularized ECM with hydrogel, which was successfully repopulated by progenitor cells derived from adipose tissue [45, 46]. This approach was used to improve heart function following infarction in a porcine MI model.

Extensive studies were also conducted on ECM isolated from decellularized pericardium. The porous nature of the material makes it suitable to cellular retention and vascularization in contrast to the ECM derived from myocardium. The scaffold is subsequently refilled with MSCs or myofibroblasts dependent on these established properties. An easily obtainable source of pericardium is from pericardium resection during surgery. The ECM is then extracted from the resected tissue for the underlying scaffold. Rejection still poses a problem, so successful implantation requires minimizing factors contributing to this phenomenon. Several studies have used this ECM scaffold to support stem cell differentiation from infiltrated cells. An in vivo study proved that decellularized pericardium refilled with MSCs patched into the myocardium for 12 weeks could improve left ventricular fraction shortening (LVFS), left ventricular systolic pressure, and support infract area vasculogenesis, cytokine secretion, and cell differentiation [47]. Another research study obtained the same results while highlighting functional enhancements in the heart pumping features [48]. Additionally, decellularized pericardium ECM scaffold modified with fibroblast growth factor (FGF) was taken into consideration for its synergistic effects in MI rats and demonstrated clear advantages in spurring angiogenesis [49].

In another study, noncardiac ECM matrix, derived from sources such as small intestine submucosa (SIS), skin, and urinary bladder, were used for the regeneration of cardiac tissue, and some protocols to this effect were approved by the FDA [16]. Decellularized urinary bladder matrix was used as an epicardial cardiac patch material, showing cellular infiltration into the patch and providing functional benefits following transplant [50, 51]. SIS was considered to have perfect biocompatibility and mechanically modifiable characteristics. The injection of SIS ECM scaffold alone into the mouse MI model illustrated preserved heart morphology, reduced the infarct area, and increased the number of blood vessels [52]. Tan et al. found that the SIS ECM could enhance the cardiac systolic and diastolic functions without any adverse immunological response [53]. Notably, the expression of cardiac troponin T and *α*-smooth muscle actin in SIS helps seeded cells differentiate into cardiac lineages, thereby accelerating the therapeutic results of this technique. Currently, a clinical trial (NCT02139189) is in progress to test the feasibility and safety of the SIS scaffold (Table 2).

Although decellularized tissues and organs can provide fibrous scaffolds that resemble the native structure and biochemical composition, providing a scaffold with specific properties for a particular patient is still a great challenge. Despite this challenge, progress has already produced great and encouraging results, while the recellularization of three-dimensional native tissues and organs remains a debated proposition. One research team sliced decellularized bovine pericardium into three-layer tissue using a cryostat microtome to build 3D structures [54]. Such scaffolds containing more than a few layers of seeded cells are crucial to creating thick and viable cardiac tissues. This approach is demonstrated in studies reporting restoration of dilated LV and preservation of cardiac functions for large infarctions from this type of cardiac patch. Blazeski et al. reported that slices of rat myocardium ECM were prepared by starting with Langendorff perfusion and sectioning into 5 to 8 mm diameter, 300 mm thick slices, which are considered as essentially 2.5-dimensional tissues [55]. After cells populate the scaffold slices, the total product behaved as an integrated, functional tissue, serving as a model system for studies of physiological and pathophysiological myocardial function in vitro.

These challenges are even more daunting in the context of whole heart tissue engineering. It was recently shown that perfusion of intact rat and porcine hearts with detergents leads to decellularized whole organ scaffolds by utilizing the native vasculature to access, lyse, and remove all of the cells. This made it possible to use the whole decellularized myocardial ECM for cardiac repair [22, 56]. At the same time, much of the work in whole heart tissue engineering focuses on using decellularized cadaveric hearts, retaining native macro- and microvasculature and ECM, structures which best promote the differentiation of seeded adipose tissue-derived stem cells (ATDSCs) and MSCs into site-specific cell populations [57, 58]. Sánchez et al. demonstrated that decellularization of cadaveric whole human hearts provided a cytocompatible scaffold with an intact 3D architecture and a preserved vascular network. Therein, the scaffold promotes cardiomyocyte gene expression in reseeded stem cells, organizes existing cardiomyocytes into nascent muscle architecture, and shows electrical coupling [22]. Yasui et al. examined the conduction properties of recellularized scaffolds of whole hearts, reporting that the hearts showed dynamic excitation-propagation as a “whole organ.” Thus, there is a strong belief that the engineered whole heart will act as an alternative for cardiac transplantation in the near future [59].

Recently, researchers have already attempted to “cross the kingdom,” using decellularized plants as perfusable tissue engineering scaffolds. Gershlak et al. reported their finding from using leaf matrix to build a decellularized cardiac scaffold, taking full advantage of the leaf veins. They used the MSCs and iPSCs derived cardiomyocytes to adhere to the outer surfaces of the plant scaffolds and demonstrated contractile function and calcium handling capabilities after 21 days of culture. This remarkable finding provides us a new route for building efficient scaffolds to produce large patches of functional vascularized tissues [60].

In summary, decellularized scaffolds play a primary role in cardiac engineering tissue formation with the advantages of superb biocompatibility, biodegradability, and the endogenous vascular structure. However, they are still limited by their material source and morphology; they are unable to transform into any shape of injured or myocardium tissue zone for replacement. With the large amount of progress made, the technique of engineering tissues from decellularized scaffolds burgeoned from fabricating small pieces to creating macropatterns. Studies presented so far have attempted to recellularize the whole heart skeleton with multiple cell types to reform a functional heart for transplant purposes, which opens more paths to effectively recreate the organ from tissue donors.

## 4. Artificial Scaffolds

### 4.1. Synthetic Natural Fibrous Matrix

Production and evaluation of synthetic fibrous scaffolds permit a higher degree of manufacturing control than tissue decellularization, chiefly by allowing manufacturers to make specific architectures according to a desired shape. This characteristic lends opportunities to design manufacturing procedures with increased reproducibility and reliability. The synthetic fibrous scaffolds are manufactured following a “bottom-up” procedure, unlike the decellularized ECM scaffolds which are always produced following a “top-down” approach [39]. According to “bottom-up” principles, the characteristics of the scaffold, such as the fiber diameter, fiber alignment, scaffold porosity for cell infiltration, and macroscopic scaffold geometry, can be controlled by simply varying the needle diameter and applying needle voltage, flow rate, or viscosity of the solution/melt, along with a number of other spinning parameters [37, 61]. Currently, the electrospinning system is the most commonly used technique for producing artificial fibers. Moreover, electrically conductive fibers and metabolic sensors have been incorporated into “smart” electrospun scaffolds for real-time tissue performance monitoring. This design is directly intended to make the matrix conform to a more natural and functional architecture [23, 37, 62]. The technique was first implemented in the 1990s, using natural material as the first choice for the foundation of the scaffold, due to considerations of biological features and possibilities of rejection of synthetic polymers., Collagen, a predominant protein in cardiac ECM, was identified as a potential source that can provide mechanical support for tissue morphology and contribute to the particular cardiac ECM microenvironment. It is biocompatible, adhesive, useful for sutures, porous, and readily integrates with other materials, ultimately making collagen an appropriate molecule for a scaffold. The injection of type I collagen and growth factor into MI models without any cell seeding was shown to prevent adverse cardiac remodeling and deterioration of heart function over a very limited time [63]. Further, collagen promotes angiogenesis and inhibits apoptosis of cardiomyocytes [64]. Collagen scaffolds combined with several growth factors, other molecules, and different cell lines were measured across a series of studies to investigate the efficacy of the scaffolds. Type I collagen scaffold modified with interleukin-10 (IL-10) and filled with MSCs proved to increase the thickness of infarcted walls and increase LVEF after delivery into injured hearts [65], an intervention which is more beneficial than with scaffold alone. ATDSCs, bone marrow MSCs, Sca-1^+^ cells, and human bone marrow mononuclear stem cells (BMMNC) were seeded on collagen scaffolds to assess the therapeutic potential in treating MI. Research studies have shown positive results of these scaffolds, reporting better LVEF, reduced infarct area, and increased neovascularization, among other beneficial effects [66–68]. The MAGNUM study was the (first?) clinical trial of collagen scaffold therapy, providing encouraging results (Table 2) [17]. However, another study determined that combination scaffolds induce more inflammation responses compared to non-cross-linked structures [65]. Furthermore, it is important to evaluate the impacts of the ratio of collagen type I and III in the implanted heart as this ratio is often correlated with heart function.

Aside from collagen, other natural molecules also exist for scaffold building. Fibrin can be obtained from the patient's blood and appears to be a smart candidate to avoid the risks of adverse immunological responses. Adjusting the fibrinogen concentrations and/or polymerization rates to modulate the matrix density, mechanical strength, and microstructure permits facile manipulation of this molecule [69, 70]. Due to its intrinsic properties, implanted fibrin patches exert the highest vessel density of natural material scaffolds [71]. Compared to collagen, many more types of cells can adapt to fibrin scaffolds, including adipose-derived MSC, ESC-derived cardiac cells, iPSCs, smooth muscle cells, and fibroblasts. Ye et al. first reported an in vivo study of fibrin scaffolds seeded with iPSC-derived cardiac cells, leading to LVEF improvement by 52% and enhancing contractility in 4 weeks [72]. Additionally, modifying architecture with thymosin *β*4 encapsulation could promote the survival of cardiomyocytes against hypoxic conditions [72, 73]. Using this molecule, a series of experiments in MI models demonstrated that fibrin patch applications benefit the prognosis of the pathological response [74]. This promising direction contributed to approving the first human clinical trial, called ESC-derived progenitors in severe heart failure (ESCORT) (NCT02057900) (Table 2).

Other molecules such as chitosan, alginate, hyaluronic acid, gelatin, and even albumin were manufactured into fibrous scaffolds with their own properties in biocompatibility, biodegradability, and capacity to combine with conductive materials for normal cardiac electronic activity. Much evidence has supported the positive effects of natural material fibrous scaffolds in treating MI. For example, chitosan is capable of high growth factor retention and strong cellular receptor adhesion due to its hydrophilicity [75, 76]. Alginate can promote the formation of myofibers and cardiac gap junctions [77]. Hyaluronic acid can play a key role in cell behavior and adhesion, wound healing of infarcted myocardium, and regulating inflammatory responses [78]. Gelatin is a type of natural polymer, supplying the advantages of low antigenicity and low cost for industrial production [79]. Recently, Fleischer et al. reported that a three-dimensional cardiac patch was fabricated from albumin fibers [80]. Compared to synthetic fibrous scaffolds, cardiac cells cultured within aligned or randomly oriented albumin scaffolds formed functional tissue, exhibiting significantly improved function early on, including a higher beating rate, and higher contraction amplitude. Moreover, several attempts were made to integrate different types of materials to improve biological function, such as chitosan/alginate, alginate/fibrin, hyaluronic acid/gelatin, and hyaluronic/silk fibrin. Each combination takes advantage of the properties of each natural material. Such combinations were shown to improve the biocompatibility, biodegradability, and electronic conduction of the resulting scaffold and tissue [28]. Although some parameters did not show a significant difference between combinatorial and noncombinatorial methods, there are still calls for more research in the future.

The synthetic natural fibrous scaffold can improve cardiac contractile function. However, the major obstacle is how to induce proper vascularization. To address this issue, several types of growth factor coating scaffolds capable of this property were established. Nillesen et al. showed acellular collagen-heparin scaffolds that contained both FGF2 and vascular endothelial growth factor (VEGF), which could promote angiogenesis and blood vessel maturation [81]. Collagen scaffolds with covalently immobilized VEGF improved tissue formation by promoting cell and vessel proliferation both in vitro and in vivo, ultimately increasing blood vessel density while reducing construct thinning in a rat model of RV free wall repair [82]. Improved and rapid vascularization of implanted grafts can help overcome the limited transport of oxygen and nutrients into the implanted tissue. To this end, collagen scaffolds with covalently immobilized VEGF have suitable mechanical and biological properties for potential use in repairing heart injuries.

Proper myocardium regeneration and function is largely dependent on the properties of the scaffolds (Table 1). Some physical stimulation is considered beneficial to the biological function of artificial synthetic fibrous scaffolds. Sapir et al. presented a novel strategy for creating a functional cardiac patch by combining the use of a macroporous alginate scaffold impregnated with magnetically responsive nanoparticles (MNPs) and the application of external magnetic stimulation [83]. As demonstrated, these natural material based scaffolds provide a robust, biocompatible, microenvironment that can recapitulate function cardiac tissue following seeding, induction of differentiation, application of growth factors, and integration of electrical and mechanical properties.

### 4.2. Artificial Synthetic Polymer Scaffold

With the rapid development of polymer materials, scientists have attempted to find another approach to the fibrous matrix for heart repair. The structure of the matrix can be better controlled from micro- to macrosynthesis by using the artificial synthetic polymer scaffold. Additionally, it creates more possibilities of architecture design with much wider material and solvent diversity than the natural fibrous materials. Over the past 10 decades, several types of polymer material were tested in vitro or in vivo, such as poly(glycolic acid) (PGA), Poly(lactic-co-glycolic) acid (PLGA), polycarbonate-urethane (PCU), Poly(glycerol sebacate) (PGS), poly(L-lactic acid) (PLLA), poly(3-hydroxybutyrate-co-3-hydroxyvalerate) (PHBHV), and PCL. Polymer fibers from these sources always provide excellent biomechanical properties and are readily transformed into the shape of the injured area. However, because they are artificial polymer scaffolds, it is impossible to stimulate the iPSCs, MSCs, or ESCs to differentiate and proliferate into cardiomyocytes with full function on the scaffolds. Thus, cell sources chosen to seed on such particular matrices include neonatal rat ventricular cardiomyocyte (NRVCM) and cardiac/smooth muscle cell lines, typically H9C2 and C2C12. Zong et al. identified that the growth of NRVCMs was controlled by fiber structural cues [84], thus imposing the requirement to develop a scaffold with the appropriate fiber properties. Kai et al. reported PCL matrix with a functionalized plasma surface attenuated dilatation in the MI model [85], and Kenar et al. demonstrated that the PCL patch reduced the scar size following MI [86]. Another article revealed that PLGA and PCU fibrous structures supported the alignment and elongation of seeded NRVCMs and H9C2 myocytes, which improved contractile synchrony [87].

Even though polymer fibers provide properties of biomechanics and contraction, they lack the capacity of electronic conduction required for the heart without other input. Thus, efforts have focused on combining natural ECM proteins with the enhanced structural stability of polymers to form so-called “biohybrid” nanofibers containing conductive materials [88, 89]. Notable results showed improved contractile synchronicity in cardiac tissues grown in porous scaffolds containing gold nanowires compared to scaffold alone [88]. More recently, Kharaziha et al. reported stronger spontaneous and synchronous beating cardiac tissue grown on aligned PGS/gelatin electrospinning nanofibrous matrix containing carbon nanotubes [89]. Taken in whole, these studies provide a vision to build more functional macromeshes to serve as substrates for bioactive myocardial patches, or even a whole heart (Table 1).

#### 4.2.1. Macropatterned Meshes

The real challenge of any synthetic fiber technology is building the global, 3D structure of the target cardiac tissue for the final solution of MI or HF therapy. Thus, the “biohybrid” or “bioartificial” approaches offer promising ways to create macrosize myocardium meshes. The meshes in turn have more capabilities to stimulate cell adhesion and proliferation, as well as better conductive characteristics to support the synchronous contraction of large-scale myocardium. Recently, several 3D scaffolds were produced with cardiac ECM structures using various bioartificial materials. Cristallini et al. fabricated PHBHV/gelatin bioartificial constructs that mimicked the anisotropic structure and mechanical properties of the myocardium, while simultaneously demonstrating their suitability in favoring cell adhesion and differentiation. The biodegradable nature suggested the ability of these matrices to drive the growth and organization of cells for a prolonged time. The scaffold made it possible to insert a patch directly in vivo during the early phase after MI. Hsiao et al. reported an aligned, conductive, nanofibrous mesh of polyaniline (PANI)/PLGA that was successfully prepared by electrospinning. After doping by HCl, the mesh was electrically conductive and carried positive charges, which is essential for the subsequent cardiomyocyte adhesion, formation of isolated cell clusters, and electrical stimulation [90].

#### 4.2.2. Biodegradable Behavior

Artificial synthetic materials offer the chance to design a material with the appropriate porosity and mechanical stability [91]. However, non-biomaterials based matrices still carry higher risks of chronic rejection, unstable biocompatibilities, and inability to heal and grow during maturation. In contrast, biodegradable scaffolds can produce suitable tissue that heals without scarring and avoid the chronic rejection response. Fujimoto et al. applied an elastic, biodegradable polyester urethane urea (PEUU) cardiac patch onto subacute infarcts, which promoted smooth muscle tissue formation, improved cardiac remodeling, and bettered contractile functioning during chronic stage pathology [92]. Variations in poly(N-isopropylacrylamide) (PNIPAAM) were developed and evaluated as injectable therapies. Basic results led to development of a series of scaffolds based on PNIPAAM, including biodegradable dextran (Dex) grafted PCL-2-hydroxyethyl methacrylate (PCL-HEMA)/PNIPAAM (Dex-PCL-HEMA/PNIPAAM), and copolymerization of NIPAAM, acrylic acid (AAc), and hydroxyethyl methacrylate-poly (trimethylene carbonate) (HEMAPTMC), to create poly(NIPAAM-co-AAc-co-HEMAPTMC), and were shown to preserve or improve cardiac function in small animal infarct models with the property of releasing molecules into the surrounding media characteristic of biodegradation [93–95]. These cell-engineered patches created from biodegradable biomaterials require extensive angiogenesis in order to transport oxygen and nutrients in vivo. This requirement supports engrafting implanted and/or recruited progenitor cells required for tissue formation as the patch degrades. The optimal method to induce and maintain vascularization within a biomaterial patch requires further investigation to make biodegradable scaffolds more functional and suitable for clinical applications.

## 5. Scaffold-Free: Orientation of Cardiac Tissue Engineering?

### 5.1. Hydrogel Technology

Hydrogel technology is the most commonly used method to produce scaffold-free cardiac tissue, relying on a minimally invasive, catheter-based approach for myocardial repair, as an alternative to direct matrix injection and tissue implantation. Currently, delivery of alginate hydrogel in rat and porcine MI models was proven to preserve heart function while minimizing left ventricular enlargement and myofibroblast proliferation [96, 97]. Early Phase I FDA-approved trials called PRESERVATION have demonstrated similar efficacy of transcoronary alginate infusion for the treatment of MI within humans, now transitioning to Phase II trials based on these positive results (Table 2) [18]. At the same time, Algisyl-LVR injection trials called AUGMENT-HF were approved by the FDA and are now progressing into Phase III to treat dilated cardiomyopathy (Table 2). However, there is not current approval for HF treatment with hydrogel and further work is necessary to expand its applications [19].

Early in 2002, Zimmermann et al. first reported an engineered hydrogel heart tissue containing collagen, a basement membrane protein mixture (Matrigel), and matrix factors that supported neonatal cardiomyocytes. Furthermore, they used casted circular molds and subjected them to phasic mechanical stretching. This process generated implanted cells displaying important hallmarks of differentiated myocardium [98]. Based on this type of matrix, they developed heart muscle constructs that fulfilled some prerequisites of graft material for therapeutic approaches, later described as self-assembled engineered heart tissue (EHT) [9, 99, 100]. EHTs are highly advantageous, displaying morphological, electrophysiological, and contractile properties similar to native heart muscle preparations, and are designed to fit various shapes and sizes matching the coronary diameter and the infarcted area [7, 100, 101]. In addition, they have excellent stability and biocompatibility as assessed by the survival features of EHTs in vivo under various conditions [102].

Another study confirmed the bioactivity of hydrogel is maintained and facilitates self-assembly of cells into natural tissue-like structure [103]. The combination of hydrogel scaffolds with human ESC-derived cardiomyocytes enhanced proliferation and creation of a muscle network demonstrating properties of a more functional cardiac tissue patch [104]. In addition, purified cardiomyocytes colonized on EHTs showed aligned, striated cardiomyocytes and created active twitch forces of up to 70 *μ*N [105]. Zhang et al. attempted to improve the electrophysiological function of cardiomyocytes attached to hydrogel scaffolds, reporting enhanced capabilities to heal large infarct areas [106]. However, a large limitation in the EHTs application is the propensity to retain the structure and biomechanics of the injured hearts with increased LVFS, rather than improvement of LVEF.

In addition, a series of experiments was conducted to mix only Matrigel and cells, mainly ESCs or MSCs. Compared to the collagen matrix, some studies of collagen-less hydrogel EHTs revealed significant improvements in LVEF, increased ventricular wall thickness, and support of diastolic function. Additionally, synthetic bioactive hydrogels, containing enzymes such as metal matrix proteases (MMPs), were created to assess potential enhancement of contractile performance. In contrast to Matrigel, the synthetic bioactive matrix has increased survival of transplanted human ESC-derived cardiomyocytes cells [107], proving highly relevant to the clinic.

Recently, artificial synthetic materials were also used to build hydrogel scaffolds. An approach based on a biodegradable hydrogel made from PEGylated-fibrinogen (PF) matrix was reported by Habib et al. [108]. These investigators optimized the PF hydrogel for an in vitro 3D culture with NRVCMs or ESC-derived cardiomyocytes. This is turn led to the development of a functional cardiac syncytium and improved cardiac function and survival after implantation (Table 1).

### 5.2. Self-Assemble Tissue with Multiple Cells

The general strategy for cardiac tissue engineering is to combine cells suitable for cardiac therapy with a scaffold or simply some hydrogel. As aforementioned, there are several approaches for cardiac tissue engineering, such as the in situ delivery of cells with injectable biomaterials, in vitro engineering of contractile tissue constructs, and the initiation of clinical trials based on these results [16, 42, 45]. However, there are some limitations that prevent matrix-based tissue engineering from moving forward. Notably, natural biologic molecules are known to cause immunogenic responses, and the mechanical properties of engineered scaffolds do not mimic those of the native myocardium [109, 110]. Additionally, artificial synthetic fibers still required further research prior to clinical transition due to the lack of sufficient biocompatibility. These problems led to the conception of self-assembled tissues with multiple cells.

Noguchi et al. used 3 cell sources (cardiomyocytes, endothelial cells, and fibroblasts) with optimal cell ratios to create a scaffold-free spheroid with 3D structure, which provides a rich extracellular matrix more conducive to the cardiac environment [111]. Furthermore, spheroids can assemble to form certain morphologies. Another group used human ESC-derived Isl1^+^ cells, human iPSCs, and MSCs to create a method of hanging a drop culture to form scaffold-free 3D microtissue based on gravity-enforced cell assembly [14]. The concept of 3D microtissues in vitro represents a promising strategy for enhancing cellular engraftment, survival, and biocompatibility.

## 6. Future Aspects

The field of cardiac tissue engineering has made several great breakthroughs in the past 15 years, expanding the potential for tissue regeneration in future human and model medicine. Although 4 types of engineered tissue are approved for clinical trials, there are still extreme challenges facing scientists. The heart is an organ with excellent biomechanical properties to support high pressure and stress. Some natural materials can only provide 70 *μ*N forces, much lower than the contractile forces of 50 mN in the native cardiac tissue under optimized preload. Moreover, cell densities within bioengineered tissues are lower than those in the native tissue, resulting in less contractile force. These limitations are severe detriments to realizing clinical application of large engineered cardiac tissue.

Vascularization is another major concern in engineering functional cardiac tissue. The high metabolic rate of cardiomyocytes necessitates a dense capillary system in the cardiac tissue [112]. Small pieces of artificial tissues are able to draw oxygen and nutrition directly from the surroundings, but larger pieces of tissues and whole organs require vascularization for survival. The decellularized scaffold is the best choice of building large size tissue currently. Vascularization of the microfabricated cardiac tissue prior to implantation provides a practical alternative. Furthermore, the combination of angiogenesis factors such as VEGF and FGF for artificial synthetic matrices displays encouraging results.

Functionality of generated organs or tissues plays a central role in vivo and in clinical applications. The success of cardiac engineered tissues depends on a bioactive scaffold, some matrices with the optimal cells, or a combination of both. During the past decade, several types of cells were modified with different stimulant, with the intent of changing properties for enhanced functionality. In parallel, genome editing greatly facilitates the development of stem cell and tissue engineering with specific allelic variants, providing invaluable research tools for powerful stem cell lines. These lines are tailored to have better adhesion, proliferation, and differentiation capabilities, contributing to the ability to efficiently layer a coated scaffold. As shown here, cardiac tissue engineering is on the way to act as a feasible alternative for MI and HF therapies.

## Figures and Tables

**Table 1:** Different method to build matrix in cardiac tissue engineering.

Type	Matrix source	Cell source	Modification	Improvements after transplantation	Advantage	Shortage	Clinical trial
Decellularized	Cadaveric and animal source: Myocardial ECM, pericardium ECM, SIS, UBM, and so on	Alone, MSC, ATDSC, NRVCM, cardiomyocytes, and so on	FGF, HGF	LVEF ↑, LVFS ↑, infarct LV wall thickness ↑, infarct zone ↓, LV end diastolic and systolic pressure improvements	Purely extracellular matrix Exact multiscale structure Excellent biocompatibility Less rejection responses Preserved vascular network	Immature cells within a mature matrix Nonuniform decellularization protocols Lack of standards for successful decellularization Variable sample composition Limited by its own architecture	CorMatrix ECM trial [16]

Fibro matrix	Natural fibers: Collagen, fibrin, chitosan, alginate, hyaluronic acid, gelatin, albumin, and so on Artificial synthetic fibers: PGA, PLGA, PCU, PGS, PLA, PCL, PEG, and so on	Natural fibers always mixed with differentiated and proliferative potential cells: iPSC, MSC, ESC, BMMNC, and so on or simply alone. Artificial synthetic fibers usually are seeded with cardiac/smooth muscle cells: NRVCM, H9C2 cell line, C2C12 cell line, and so on	VEGF, FGF, HGF, IGF, TGFb, SDF-1a, physical stimulation, etc. “bio-hybrid”	Cell survival and retention ↑, LVEF ↑, LVFS ↑, contractile synchronicity ↑, LV end-diastolic pressure ↓, LV pressure change ↑, infarct size ↓, fibrosis ↓	Diversity of materials and solvents Control of fiber morphology (nano to macro) Nano-micro scale fiber fabrication Prefect force strength Well conduction velocity	Requires conductive polymers and solvents Low production rates Reproducible fiber production requires environmental control Less biocompatibility Lack of native stimulations for cell proliferation Considerate biodegradable behavior	MAGNUM [17] ESCORT [NCT02057900]

Hydrogel tissue model	Matrigel, Collagen, and so on	Alone, ESC, NRVCM, myoblasts, cardiomyocytes, and so on	VEGF, FGF	LVFS ↑, infarct size ↓, infarcted/noninfarcted wall thickness ratio ↑, LV wall thickness preservation Usually no LVEF improvements without components	Scaffold free Minimally invasive Catheter-based approach available	Limited to build macropieces Persist heart pressure but not improve contractile function among all researches	PRESERVATION [18] AUGMENT-HF [19]

ATDSC: adipose tissue derived stem cell; BMMNC: bone marrow mononuclear cell; ECM: extracellular matrix; ESC: embryonic stem cell; FGF: fibroblast growth factor; HGF: hepatocyte growth factor; IGF: insulin-like growth factor; iPSC: induced pluripotent stem cell; LVEF: left ventricular ejection fraction; LVFS: left ventricular fractional shortening; MSC: mesenchymal stem cell; NRVCM: neonatal rat ventricular cardiomyocyte; PCL: poly(*ε*-caprolactone); PCU: polycarbonate-urethane; PEG: polyethylene glycol; PGA: poly(glycolic acid); PGS: poly(glycerol sebacate); PLA: polylactic acid; PLGA: poly(lactic-co-glycolic) acid; SDF-1: stromal cell derived factor-1; SIS: small intestine submucosa; TGF: transforming growth factor; UBM: urinary bladder matrix; VEGF: vascular endothelial growth factor.

**Table 2:** Summary of clinical trials quantitative data using various scaffold in cardiac tissue engineering.

Study name	Scaffold	Objective	Patients	Diagnosis	Pretreatment heart function	Surgical procedure	Posttreatment heart function	Follow-up time	Adverse impacts
CorMatrix ECM trial	CorMatrix (decellularized porcine small intestinal submucosa)	To evaluate the safety of CorMatrix for intraventricular repair of mechanical complications of MI	11 consecutive patients Between July 2011 and October 2012 Age 67 ± 11 years	LV aneurysm, Ischemic VSD, MI	LVEF 31 ± 7%	All the patients underwent patch repair using CorMatrix ECM with a running Prolene suture technique	The data of LVEF not provided; No evidence of ventricular thrombus, and there were no thromboembolic events	207 ± 211 days	No complications of CorMatrix ECM repair failure including readmission for any cardiac cause or death

MAGNUM	Collagen matrix	To evaluate intrainfarct cell therapy associated with a cell-seeded collagen scaffold grafted onto infarcted ventricles	15 consecutive patients Aged 54.2 ± 3.8 years	MI with surgical indication for CABG and LV wall has postischemic scars	NHYA FC 2.3 ± 0.5 LVEF 25 ± 7% LVEDVol 142 ± 24 ml LVFDT 162 ± 7	3D collagen matrix seeded with the BMCs was added on top of the scarred area at the end of surgery after BMCs injected into the same area	NHYA FC 1.4 ± 0.3 (*p* = 0.005) LVEF 33 ± 5% (*p* = 0.04) LVEDVol 117 ± 21 ml (*p* = 0.03) LVFDT 196 ± 8 (*p* = 0.01) Blind Radioisotopic/MRI showed that 58 ± 9.3% of the cell-implanted segments improved their kinetics and viability	3 months	Not reported

ESCORT (NCT02057900)	Fibrin patch matrix	To assess the feasibility and safety of a transplantation of cardiac-committed progenitor cells	Patients recruiting (estimated enrollment 6 patients)	Ischemic heart disease	NHYA FC and LVEF	Add a fibrin gel embedding hESCs-derived CD15^+^ Isl-1^+^ progenitors in addition to CABG and/or a mitral valve procedure	Plan to measure feasibility of patch's generation and its efficacy on cardiac functions	Within 1 year	To record clinical/biological abnormalities including arrhythmias

PERSERVATION (NCT01226563)	IK-5001 (an injectable, bioabsorbable scaffold)	To test the feasibility of intracoronary delivery bioabsorbable scaffold to prevent adverse left ventricular remodeling and dysfunction	27 patients Age 54 ± 9 years	Moderate-to-large MI	Minnesota Score LVEF NT-proBNP 2977 ± 5392	To place an infusion catheter immediately distal to the deployed stent and 2 ml IK-5001 was injected into the IRA	At the end point (180 days) of observation Minnesota Score 16 ± 3 (*p* < 0.05 compared with day 30) NT-proBNP 566 ± 847 (*p* < 0.05)	180 days	No significant ventricular arrhythmia was observed; None of adverse events were judged to be related to the device

AUGMENT-HF (NCT00847964)	Algisyl-LVR (self-gelling alginate hydrogel)	To measure a tissue engineering strategy to increase wall thickness and reduce chamber diameter	6 patients	Dilated cardiomyopathy	LVEF 28.7 ± 8.5% LVEDV 139.5 ± 20.6 ml LVESV 99.8 ± 25.8 ml KCCQ score 39.4 ± 28.0 Number of patients in NYHA class III/IV: 6	All the patients received left ventricular restoration with 10–15 implants of Algisyl-LVR concomitant with coronary artery bypass or valve surgery	LVEF 36.0 ± 13.5% LVEDV 123.6 ± 18.6 ml LVESV 77.2 ± 29.5 ml KCCQ score 74.0 ± 25.0 (*p* < 0.05) No. of patients in NYHA class III/IV: 1	3 months	No significant cardiac adverse events were recorded

MI: myocardial infraction; LVEF: left ventricular ejection fraction; LVEDV: left ventricular end diastolic volume; LVESV: left ventricular end systolic volume; VSD: ventricular defect defect; NHYA FC: New York Heart Association functional classification; LVFDT: left ventricular filling deceleration time; IRA: infract-related artery; KCCQ: Kansas City Cardiomyopathy Questionnaire.
